# Attachment, mentalisation and expressed emotion in carers of people with long-term mental health difficulties

**DOI:** 10.1186/s12888-018-1842-4

**Published:** 2018-08-16

**Authors:** Mary Gemma Cherry, Peter James Taylor, Stephen Lloyd Brown, William Sellwood

**Affiliations:** 1 Division of Clinical Psychology, University of Liverpool, Whelan Building, Quadrangle, Brownlow Hill, Liverpool, L69 3GB UK; 2 Division of Psychology & Mental Health, University of Manchester, Zochonis building, Manchester, M13 9PL UK; 3 Department of Psychological Sciences, University of Liverpool, Whelan Building, Quadrangle, Brownlow Hill, Liverpool, L69 3GB UK; 4 Division of Health Research, University of Lancaster, Furness Building, Bailrigg, Lancaster, LA1 4YW UK

**Keywords:** Attachment, Mentalisation, Expressed emotion, Carers, Cross-sectional, Quantitative, Criticism, Hostility, Emotional over-involvement

## Abstract

**Background:**

Expressed emotion (EE) is a global index of familial emotional climate, which is comprised of emotional over-involvement (EOI) and critical comments (CC)/hostility. Although EE is an established predictor of negative outcomes for both people with long-term mental health difficulties and their family carers, its psychological underpinnings remain relatively poorly understood. This paper examined associations between attachment, mentalisation ability and aspects of EE.

**Methods:**

Carers of people with long-term mental health difficulties (*n* = 106) completed measures of adult attachment (the Experiences in Close Relationships-Short Form questionnaire), mentalisation (the Reading the Mind in the Eyes Test and the Emotional Self-Efficacy Scale) and EE (the Family Questionnaire). Data were analysed using hierarchical multiple regression.

**Results:**

Attachment avoidance and facets of mentalisation were directly and uniquely positively associated with CC/hostility, with attachment avoidance and other-directed emotional self-efficacy (one facet of mentalisation) each significantly predicting CC/hostility scores after controlling for the effects of EOI and demographic variables. However, no associations were observed between EOI, attachment anxiety and mentalisation. Furthermore, no indirect effects from attachment to EE via mentalisation was found.

**Conclusions:**

Although it would be premature to propose firm clinical implications based on these findings, data indicate that it may be beneficial for clinicians to consider attachment and mentalisation in their conceptualisation of carers’ criticism and hostility. However, further research is needed to clarify the magnitude of these associations and their direction of effect before firm conclusions can be drawn.

## Background

Approximately six and a half million people in the United Kingdom (UK) provide unpaid care to others, typically family members or close friends, with this number projected to rise to nine million by 2037 [1]. Of these, approximately 13% (equivalent to one in 10 people in the UK) provide care to someone with a long-term mental health difficulty, saving the UK economy an estimated 17 billion per year [2]. Caring for someone with a long-term mental health difficulty can be a challenging and emotional experience, with carers often displaying higher levels of anxiety, depression, and general psychological distress than members of the general population [3, 4]. As such, a strong moral and financial argument can be made for developing effective, flexible and inclusive services and interventions which support carers in their roles and safeguard their wellbeing, and the wellbeing of those to whom they provide care [5].

Over the last 60 years, increasing research attention has been paid to the potential influence of family environment on care outcomes, particularly the role of ‘expressed emotion’ (EE). The term ‘expressed emotion’ encompasses particular attitudes, emotions and behaviours expressed by family carers towards the person (s) to whom they provide care [6]. Key components include emotional over-involvement (EOI), critical comments (CC), and hostility [6]. Emotional over-involvement is characterised by overly self-sacrificing and/or intrusive behaviours and exaggerated emotional responses, whereas the term ‘CC/hostility’ is commonly used to refer to critical, negative or blaming attitudes or statements towards service-users [6].

Expressed emotion is a complex concept which can evoke in family carers immense guilt and shame [7], which in turn can influence EE behaviours [8]. Whilst not pathological in itself, EE is a consistent and reliable predictor of relapse across a range of mental health difficulties [9–11]. However, the psychological processes associated with EE require further understanding [6]. The majority of existing research in this area has investigated the utility of an attribution-based framework [3, 6], which postulates that EE results from carers’ appraisals of, and beliefs about, the controllability, stability and internality/externality of service-users’ mental health difficulties, rather than the specific symptomatology displayed. However, although this model has received empirical support, particularly regarding the hypothesised associations between attributions and CC/hostility [6], inconsistent findings regarding the relationship between carers’ attributions and their EOI suggest that it does not adequately account for the psychological processes underlying EOI [6].

More recently, EE has begun to be seen as a developmentally-based process of adaptation to, and coping with, illness-based separation and loss, which has led to increasing recognition of the potential application of attachment theory, and the related theory of mentalisation, to understanding individual differences in carers’ EE [7, 12]. Attachment theory is a way of understanding psychosocial development, which posits that individuals form enduring patterns of interpersonal behaviour through internalisation of interactions with their primary carer (s) in infancy [13]. These patterns are represented cognitively in the form of a stable internal working model (IWM) of attachment, which subsequently influences behaviour in close relationships throughout the lifespan, particularly those in which an individual is required to give or receive care [13]. Carers’ attachment may therefore aid or impede their ability to provide effective and attuned care [14, 15].

Carers high on attachment anxiety (characterised by habitual preoccupation and over-involvement in close relationships combined with fear of abandonment) may engage in emotionally over-involved behaviours in an attempt to facilitate interpersonal closeness [16]. Alternatively, if carers high on attachment anxiety perceive their relative to be unavailable or rejecting, they may defend against the associated painful feelings of self-blame by externalising blame onto others in the form of criticism or hostility [17]. In contrast, carers high on attachment avoidance (characterised by difficulty in trusting others, devaluation of close relationships and avoidance of intimacy) may engage in regulatory, anger-driven behaviour such as criticism and hostility in an attempt to avoid and/or cope with the discomfort associated with the caring role [18, 19].

Attachment theory may therefore provide a theoretical framework for understanding individual differences in carers’ EE [20]. It is generally accepted that attachment representations are relatively stable across an individual’s lifespan [21]. Although there is evidence to support the notion that attachment representations can be modified during psychological therapy, such change is likely to require substantial time, effort and commitment [22]. A developmental and malleable factor related to attachment style, which is also likely to be an important contributor to the development and maintenance of EE but which can be easily modified, is an individual’s ability to mentalise [23].

The term ‘mentalisation’ shares conceptual overlap with constructs such as theory of mind, emotional self-efficacy and reflective functioning [23], and can be broadly defined as the process by which an individual is able to use learned self-other representations to attend to the implicit and explicit subjective mental states and mental processes of self and others [24]. Mentalisation develops partly as a function of attachment [24]; reflective, sensitive and attuned early caregiving (i.e. relationships low in attachment avoidance and attachment anxiety) is hypothesised to facilitate well-developed mentalisation, whilst poorly attuned or neglectful early caregiving is theorised to lead to impaired mentalisation [23].

Well-developed mentalisation may help to facilitate accurate evaluation and regulation of one’s own and others’ thoughts, feelings and behaviours, and thus discourage emotionally over-involved, critical or hostile caregiving. Conversely, less well-developed mentalisation may contribute to high EE by limiting carers’ awareness of both the amount of support needed by the service-user *and* the impact of their behaviours on the service-user [24]. Mentalisation theory may therefore also form a theoretical framework for the study of individual differences in carers’ EE.

**Fig. 1:**
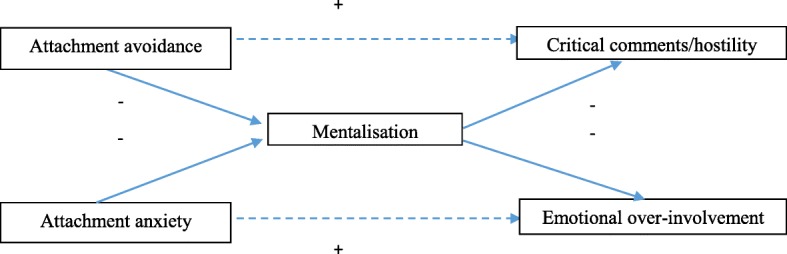
Figurative summary of mediational hypotheses

Given that mentalisation is amenable to therapeutic intervention [23], consideration of the potential effect of mentalisation on the hypothesised associations between attachment and EE may have greater clinical implications than consideration of attachment alone. However, the relationships between attachment, mentalisation and components of EE in carers of people with long-term mental health difficulties have yet to be studied. This study tested a mediational model suggesting that adult attachment dimensions differentially influence aspects of EE through their effects on mentalisation (Fig. 1; [14, 15, 17]). It is hoped that a better understanding of the relationships between these constructs will contribute to improved outcomes for both individuals with long-term mental health difficulties and their carers. Specifically the following hypothesises were explored:

1. Attachment avoidance and attachment anxiety would be positively related to CC/hostility and EOI respectively;
2. Mentalisation would be negatively related to attachment avoidance, attachment anxiety, CC/hostility and EOI;
3. Mentalisation would partially mediate the effect of attachment anxiety and attachment avoidance on EOI and CC/hostility respectively.

## Methods

### Research design

This study used a cross-sectional design with a convenience sample, using multiple self-report measures.

### Participant characteristics

Family carers of people with long-term mental health difficulties participated in this study. Inclusion criteria were that participants completed at least one of the study’s measures, and self-certified as fulfilling the inclusion criteria outlined in the participant information sheet. Specifically, participants were required to: a) be 18 years of age or over; b) provide at least 10 h of face-to-face care per week to a relative with a non-organic long-term mental health difficulty for at least 6 months; c) understand English sufficiently to provide informed consent to participate. ‘Long-term mental health difficulty’ was defined in the participant information sheet as a severe and enduring mental health difficulty, present for at least 6 months, which impairs psychological well-being and social, occupational and/or interpersonal functioning [25]. Specific mental health diagnoses were not specified as inclusion/exclusion criteria as EE influences outcome across a range of diagnoses [9]. Carers of people with organic mental health difficulties, such as learning disabilities, dementia or acquired brain injuries, were excluded.

The final sample comprised 106 carers. Participants were primarily White British (*n* = 77; 72.64%) and female (*n* = 86; 81.13%), with a mean age of 47.13 (*SD* = 13.49, range 22–87). Participants cared for relatives aged between 18 and 92 (*M* = 42.76, *SD* = 17.64), and had done so for an average of 11.46 years (*SD* = 9.66, range 1–45). Participants reported caring for individuals with a range of mental health difficulties, of which the most common were affective disorders (*n* = 79, 74.53%). Amount of care provided ranged from less than 15 h (*n* = 18, 16.98%) to over 75 h (*n* = 29, 27.36%) per week. Half of the sample (*n* = 52, 49.06%) reported caring for individuals with additional physical health, substance misuse, and/or attentional/neurological additional difficulties. Table 1 displays demographic information for the final sample.

**Table 1:** Demographic data (*n* = 106)

Demographic Variable	*n* (%) unless otherwise stated	
	Carers	Service-users
Age (years), *M* (*SD*), range	47.13 (13.49), 22-87^a^	42.76 (17.64), 18–92^b^
Gender		
Male	19 (17.92)	57 (53.77)
Female	86 (81.13)	44 (41.51)
Not stated	1 (0.94)	5 (4.72)
Ethnicity		
Caucasian	94 (88.68)	96 (90.57)
South Asian	4 (3.77)	3 (2.83)
Other Asian background	1 (0.94)	2 (1.89)
Mixed background	1 (0.94)	1 (0.94)
Other	4 (3.77)	3 (2.83)
Not stated	2 (1.89)	1 (0.94)
Employment status		
Employed	63 (59.43)	22 (20.75)
Not currently in paid employment	15 (14.15)	47 (44.34)
Student	2 (1.89)	8 (7.55)
Retired	15 (14.15)	17 (16.04)
Other	10 (9.43)	8 (7.55)
Not stated	1 (0.94)	4 (3.77)
Relationship to service-user^e^		
Partner/spouse	35 (33.02)	n/a
Parent	8 (7.55)	n/a
Child	13 (12.26)	n/a
Other	5 (4.72)	n/a
Not stated	45 (42.45)	n/a
Weekly care provision (hours)		
10–14	18 (16.98)	n/a
15–29	21 (19.81)	n/a
30–44	17 (16.04)	n/a
45–59	2 (1.89)	n/a
60–74	5 (4.72)	n/a
≥75	29 (27.36)	n/a
Not stated	14 (13.21)	n/a
Duration of caregiving (years), *M* (*SD*), range	11.46 (9.66), 1-45^c^	n/a
Duration of difficulties (years), *M* (*SD*), range	n/a	12.76 (10.91). 1-50^d^
Diagnosis, *n* (%)		
Affective disorder only	n/a	56 (52.83)
ED only	n/a	6 (5.66)
SSD only	n/a	16 (15.09)
PD only	n/a	2 (1.89)
Affective disorder and SSD	n/a	8 (7.55)
Affective disorder and PD	n/a	5 (4.72)
Affective disorder and ED	n/a	10 (9.43)
Not stated	n/a	3 (2.83)
Additional comorbid difficulties, *n* (%)	n/a	
None	n/a	48 (45.28)
Physical health difficulties	n/a	44 (41.51)
Substance misuse difficulties	n/a	1 (0.94)
Attentional/neurological difficulties	n/a	4 (3.77)
Physical health and attentional/neurological difficulties	n/a	1 (0.94)
Substance misuse and attentional/neurological difficulties	n/a	2 (1.89)
Not stated	n/a	6 (5.66)

Note: all information provided by carers

*ED* eating disorders, *M* mean, *n/a* not applicable, *PD* personality disorders, *SD* standard deviation, *SSD* schizophrenia spectrum disorders

^a^*n* = 105

^b^*n* = 104

^c^*n* = 105

^d^*n* = 101

^e^relationship data were not available for 45 participants due to an online data collection error

### Measures and covariates

#### Demographic information

A 15-item self-report measure was used to gather relevant demographic information, including information pertaining to the nature and duration of the caring role.

#### Expressed emotion

Expressed emotion was assessed using the 20-item Family Questionnaire [26]. This measure was chosen because it is the only self-report measure of EE with consistently comparable sensitivity and specificity to the Camberwell Family Interview (CFI), the ‘gold-standard’ measure of EE [26]. Participants rate the extent to which they identify with a range of statements concerning the family environment (e.g., “It’s hard for us to agree on things”) using a 4-point Likert scale. Responses produce two subscale scores: EOI and CC/hostility. Each range from 0 to 40, with low scores representing low EOI and/or CC/hostility. Participants can also be dichotomised into high or low EOI and/or CC/hostility categories based on cut-off scores of 27 and 23 respectively. The FQ demonstrates good 2-week test-retest reliability and strong internal consistency (all Cronbach’s α > .79) [26], with categories correlating highly with those from the CFI [27]. Cronbach’s α for the EOI and CC/hostility subscales in this sample were .80 and .69 respectively.

#### Attachment

Adult attachment was assessed using the 12-item Experiences in Close Relationships: Short Form (ECR-SF) questionnaire [28]. This was selected because it has favourable psychometric properties, is short in length and allows for precise and psychometrically-robust assessment of attachment [29]. Participants rate the extent to which each item describes their feelings about close relationships in general (e.g. “My desire to be very close sometimes scares people away”) using a 7-point Likert scale. Responses produce two subscale scores, attachment avoidance and attachment anxiety, which correspond to the two-dimensional model of adult attachment [29]. Each range from six to 42, with low scores indicating low attachment avoidance and/or attachment anxiety. The ECR-SF demonstrates acceptable construct validity with the original ECR, and displays good internal consistency and 6-month test-retest reliability (all Cronbach’s α > .78) [28]. Cronbach’s α for the attachment avoidance and attachment anxiety subscales in this sample were .74 and .73 respectively.

#### Mentalisation

The Reading the Mind in the Eyes Test: Revised Version (RMET) [30] and the Emotional Self-Efficacy Scale (ESES) [31] were selected to assess different aspects of mentalisation: theory of mind (ToM) and emotional self-efficacy respectively.

##### The reading the mind in the eyes test

Originally developed as a tool to discriminate adults with Asperger syndrome or high-functioning autism from controls, the RMET [30] is now widely used to assess ToM (the ability to conceive of and determine others’ mental states). It was chosen for use in this study because it is the only validated test of the extent to which individuals can identify external aspects of emotion in others that demonstrates no correlation with general intelligence [32]. Participants are presented with 36 photographs of the facial region around the eyes and are asked to choose one of four single-word descriptors of possible mental states. Scores range from zero to 36, with higher scores indicating greater ToM ability. Variable psychometric properties have been reported for the RMET; some studies have shown uni-dimensionality with good internal consistency and test-retest reliability (Cronbach’s α > .80), whilst others have found multiple factors to underlie the construct [33]. The Cronbach’s α for current sample was .58.

##### The emotional self-efficacy scale

Emotional self-efficacy was assessed using the 32-item self-report ESES. This measure was chosen because it is formulated against an established model of mentalisation (emotional intelligence) and allows for reliable and valid assessment of an important facet of mentalisation: self-perceived emotional competency in relation to self and others [34]. Participants rate their confidence in carrying out the function described by each item on a 5-point Likert scale. When scored using Dacre Pool and Qualter’s [34] revised scoring system, responses produce four subscale scores: 1) Using and Managing One’s Own Emotions; 2) Identifying and Understanding One’s Own Emotions; 3) Dealing with Others’ Emotions; and 4) Perceiving Others’ Emotions through Body Language and Facial Expressions [31]. This four-factor structure has been supported, with each factor demonstrating good internal consistency (Cronbach’s α > .80) [34]. Cronbach’s α for the four subscales in the current sample were .92, .89, .90 and .83 respectively.

### Procedure

Potential participants were invited to read the participant information sheet and complete a consent form and the study measures either online, via the Qualtrics platform (www.qualtrics.com), or by completing and returning a questionnaire pack using the stamped addressed envelope provided.^1^ Participation took approximately 20 min, and was voluntary. As an incentive, participants were offered entry into a prize draw for one of three £50 UK high street vouchers upon completion; contact details were stored separately from other data to protect participants’ anonymity.

Advertisements containing a link to complete the study online were placed on social media and UK mental health charities’ websites, Facebook pages and Twitter feeds. Twenty questionnaire packs were distributed to potential participants directly by carer support coordinators working for specialist local independent sector carer support organisations within the UK. A further ten packs were given to potential participants by the author directly, during her attendance at 4 monthly carer meetings in the North West of England (informal fora for carers to meet and share their experiences).

**Fig. 2:**
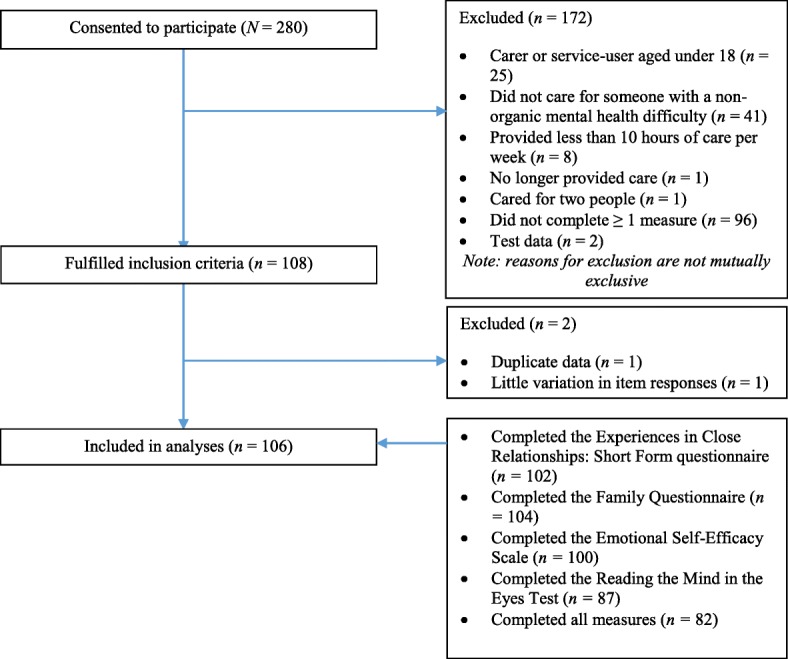
Flowchart of participant inclusion

Seven questionnaire packs were returned, and a further 273 people consented to participate online (*N* = 280), of which 108 (38.57%) fulfilled the eligibility criteria. Two were excluded (one showed little variance in their responses and one participated twice), resulting in a final sample size of 106 (37.86%; Fig. 2).

### Sample size, power and precision

A priori power calculations indicated that, in order to adequately detect a medium effect size (*f*^2^ = .15) with a.80 power level and a standard α level of.05 [35], a minimum of 104 participants were required for the most complex planned analysis: a multiple linear regression containing three control variables and the combined effect of seven predictor variables [36].

### Data analysis

All statistical analyses were conducted using the Statistical Package for the Social Sciences (SPSS) 22.0.1 [37]. Raw data were first screened for inputting errors and summed scale scores were calculated where appropriate. Scales with more than 10% of items missing were excluded from analyses (*n* = 2). As Little’s test suggested that missing data were missing completely at random (χ^2^ = 238.21, *df* = 342, *p* > .05) [38], listwise deletion was employed throughout subsequent analyses. Independent sample *t*-tests, Mann Whitney *U* tests, chi-squared tests, Analysis of Variances (ANOVAs) and correlational analyses were used as appropriate for initial data exploration, including assessment of multicollinearity between independent variables. The hypothesised associations among key variables were initially tested using correlational analyses. A series of hierarchical multiple regression analyses were then conducted to examine the hypotheses that attachment and mentalisation would each be predictive of facets of EE. Finally, potential mediation of any relationships between attachment and EE variables by mentalisation variables was tested using bias-corrected and accelerated bootstrapping over 5000 resamples with sample replacement [39].

## Results

### Initial data exploration

Table 2 displays descriptive statistics and zero-order correlations for key variables. Service-users’ age was significantly negatively correlated with CC/hostility (*r* = −.28, *p <* .01). Furthermore, females scored significantly higher than males on total EE (*M* = 58.61, *SD* = 8.34 and *M* = 51.37, *SD* = 6.35 respectively, *t*(101) = − 3.56, *p* < .01), EOI (*M* = 28.79, *SD* = 4.91 and *M* = 24.68, *SD* = 4.58 respectively, *t*(101) = − 4.10, *p* < .01) and CC/hostility (*M* = 29.82, *SD* = 4.21 and *M* = 26.68, *SD* = 3.85 respectively, *t*(101) = − 3.14, *p* < .01). All Cohen’s *d* values exceeded.80, indicating a large effect size (Cohen, 1988). No other significant differences were noted between key variables as a function of any of the demographic variables measured (all *p* values > .05). As expected, both FQ and ESES subscale scores were significantly inter-correlated. However, no significant associations were noted between RMET scores and ESES subscale scores (all *p* values > .05).

### Preliminary hypothesis testing

As shown in Table 2, attachment avoidance was significantly positively correlated with total EE and CC/hostility scores, and significantly negatively correlated with RMET scores. Furthermore, CC/hostility was significantly negatively correlated with RMET scores and borderline significantly positively correlated with E3 scores (*p* = .06). Neither EOI nor attachment anxiety were significantly correlated with any other variable.

**Table 2:** Descriptive and bivariate statistics

Variable		*M* (*SD*), range	1	2	3	4	5	6	7	8	9	10
1	Total EE	57.19 (8.45), 38–75	–									
2	EOI	28.00 (5.07), 17–38	.92**	–								
3	CC/hostility	29.19 (4.31), 20–38	.89**	.63**	–							
4	Attachment avoidance	19.80 (7.01), 6–40	.33**	.22	.40**	–						
5	Attachment anxiety	21.70 (7.29), 6–36	.16	.11	.17	.15	–					
6	RMET	25.09 (3.96), 13–34	−.20	−.15	−.23*	−.31**	−.02	–				
7	E1	30.42 (8.96), 10–50	−.03	−.07	.02	−.03	−.12	.03	–			
8	E2	20.56 (5.51), 6–30	.11	.11	.09	−.09	−.09	.18	.67**	–		
9	E3	27.41 (6.85), 8–40	.09	.03	.21	.04	−.10	−.05	.75**	.58**	–	
10	E4	9.88 (3.19), 3–15	.10	.02	.14	−.01	−.03	−.02	.70**	.67**	.73**	–

Note: *n* = 82 (correlational analyses); italicised values indicate Pearson’s product-moment correlation coefficient; non-italicised values indicate Spearman’s Rho values

*CC* critical comments, *E1* Using and managing your own emotions subscale, *E2* Identifying and understanding your own emotions subscale, *E3* Dealing with emotions in others subscale, *E4* Perceiving emotion through facial expression and body language subscale, *ECR:SF* Experiences in Close Relationships: Short Form, *EE* Expressed Emotion, *EOI* emotional over-involvement, *ESES* Emotional Self-Efficacy Scale, *FQ* Family Questionnaire, *M* mean, *RMET* Reading the Mind in the Eyes Test, *SD* standard deviation

* = significant at *p* < .05; ** = significant at *p* < .01

### Primary hypothesis testing

As EOI was not significantly correlated with any of the independent variables, no further analyses were conducted with EOI as a dependent variable. However, given the significant associations between CC/hostility and the independent variables noted above, a series of hierarchical multiple regression analyses were conducted to examine the hypotheses that attachment avoidance and mentalisation would each be predictive of CC/hostility scores. Given the results of the preliminary analyses, and the strong positively correlation noted among EOI and CC/hostility (*r* = .63), gender, EOI and service-users’ age were entered as control variables into Step 1. The independent variables were then entered into Step 2 (Table 3). The fit of data within the assumptions of multiple linear regression was assessed by examining the distribution and heteroscedasticity of regression residuals; no violations were identified. Predictor variables had variance inflation factor (VIF) factors of > .10 and Tolerance values of < 10, indicating no violation of multicollinearity assumptions. One outlier was identified (standardised residual of > 3.3). However, this was not removed and results remained the same with or without its inclusion (Cook’s distance > 1; Mahalanobis distance < critical χ^2^ value).

**Table 3:** Hierarchical multiple linear regression models showing predictors of CC/Hostility

	Variable	Cumulative			Simultaneous		
		R^2^	∆R^2^	*F* change	B	β	95% CI for B
Model 1: Demographic Characteristics, Attachment and Mentalisation as Predictors of CC/Hostility (*n* = 82)							
Step 1	Carers’ gender	.44	.42	*F* (3, 81) = 20.77**	−.58	−.06	−2.18 to 1.03
	Service-users’ age				−.05	−.24**	−.09 to −.01
	EOI				.41	.51**	.27 to.54
Step 2	Attachment anxiety	.60	.54	*F* (10, 81) = 10.51**	.02	.03	−.07 to.10
	Attachment avoidance				.11	.20**	.02 to.21
	RMET				−.10	−.09	−.28 to.08
	E1				−.12	−.26	−.24 to.00
	E2				−.05	−.07	−.22 to.12
	E3				.16	.28*	.01 to.32
	E4				.27	.22	−.08 to.61
Model 2: Service-Users’ Age, EOI, Attachment Avoidance and Understanding Others’ Emotions as Predictors of CC/Hostility (*n* = 94)							
Step 1	Service-users’ age	.38	.37	*F* (2, 93) = 27.74**	−.04	−.16	−.08 to.00
	EOI				.45	.54**	.31 to.59
Step 2	Attachment avoidance	.45	.43	*F* (4, 93) = 18.22**	.12	.20**	.03 to.22
	E3				.11	.18*	.02 to.21

Note: * *p* < .05; ** *p* < .01; *CC* critical comments, *CI* confident interval, *E1* Using and managing your own emotions subscale, *E2* Identifying and understanding your own emotions subscale, *E3* Dealing with emotions in others subscale, *E4* Perceiving emotion through facial expression and body language subscale, *EOI* emotional over-involvement, *RMET* Reading the Mind in the Eyes Test

The control variables (EOI, service-user age, carer’s age) collectively predicted a significant proportion (42%) of the variance in CC/hostility (Table 3; adjusted *R*^*2*^ = .42, *F*(3, 81) = 20.77, *p* < .01, *f*^2^ = 0.72). Inclusion of the independent variables accounted for a further 12% of the variance in CC/hostility (adjusted *R*^*2*^ = .54, *F*(10, 81) = 10.51, *p* < .01, *f*^2^ = 1.17), with service-users’ age (β = −.24, *p <* .01), EOI (β = .51, *p <* .01), attachment avoidance (β = .20, *p* < .01) and the ‘Dealing with Others’ Emotions’ subscale of the ESES (β = .28, *p* < .05) each making significant contributions to the final model. Similar findings emerged when a trimmed model (Model 2; Table 3) was estimated; the model explained a significant proportion of the variance in CC/hostility (adjusted *R*^*2*^ = .43, *F*(4, 93) = 18.22, *p <* .01, *f*^2^ = 0.75), with EOI (β = .54, *p <* .01), with attachment avoidance (β = .20, *p* < .01) and the ‘Dealing with Others’ Emotions’ subscale of the ESES (β = .18, *p* < .05) each significantly contributing. Attachment avoidance and the ‘Dealing with Others’ Emotions’ subscale of the ESES remained significant predictors of CC/hostility when the control variables were removed (β = .30, *p* < .01 and β = .22, *p* < .05, respectively), and collectively accounted for 12% of the variance in CC/hostility scores (adjusted *R*^*2*^ = .12, *F*(2, 95) = 7.33, *p* < .01, *f*^2^ = 0.14).

A post-hoc sensitivity analysis was conducted in which EOI was removed from the model. The final model explained a significant proportion of the variance in CC/hostility (adjusted *R*^*2*^ = .46, *F*(4, 93) = 6.08, *p <* .01). Attachment avoidance remained a significant predictor of CC/hostility (β = .28, *p* < .01), but the ‘Dealing with Others’ Emotions’ subscale of the ESES became non-significant, though a trend was still apparent (β = .15, *p =* .10).

#### Potential mediation of any relationships between attachment and EE variables by mentalisation variables

Table 4 shows the total indirect effect attributable to the five mentalisation variables. No evidence of indirect effects was found. To avoid Type 1 errors, we did not examine the mediating effects of the five mentalisation variables separately.

**Table 4:** Bootstrapping estimates of the total indirect effects of mentalisation variables on the relationships between attachment variables and expressed emotion variables

	Corrected estimate	SE	Lower 95%	Higher 95%
Anxious Attachment - EOI	.0076^a^	.0479	−.0918	.1095
Anxious Attachment - CC/Hostility	−.0168	.0401	−.1016	.0574
Avoidant Attachment - EOI	.0213	.0330	−.0517	.0819
Avoidant Attachment - CC/Hostility	.0213	.0230	−.0493	.0831

^a^ Figures are unstandardized beta estimates

## Discussion

This study is the first investigation known to us of the relationships among attachment, mentalisation, EOI and CC/hostility in carers of people with long-term mental health difficulties. A key contribution of the current study is the finding that, in a carer population, both attachment avoidance and facets of mentalisation were directly, and independently, positively associated with self-reported CC/hostility even after controlling for EOI. However, data indicated no support for the hypothesised relationships between attachment anxiety, mentalisation and EOI. Furthermore, there was no support for the hypothesis that adult attachment dimensions would differentially influence aspects of EE through their effects on mentalisation ability.

As predicted, avoidantly attached carers were less able to detect external explicit aspects of others’ emotional states (i.e. have less well-developed mentalisation) and were more likely to report engaging in critical or hostile caregiving behaviours than their counterparts [18, 24]. This supports the notion that carers high on attachment anxiety may behave in a critical or hostile way in an attempt to regulate their discomfort with the close caregiving role [13]. However, the hypothesis that facets of mentalisation would be negatively associated with CC/hostility was only partially supported. As expected, a significant negative correlation was noted between RMET and CC/hostility scores. However, RMET scores did not significantly predict CC/hostility scores after controlling for the effects of EOI, gender, and service-users’ age, thereby militating against considering mentalisation, as assessed using the RMET, as a significant contributor to CC/hostility. Furthermore, ESES ‘Dealing with Others’ Emotions’ subscale scores significantly and independently positively predicted CC/hostility scores when EOI was controlled for in a regression model, indicating that carers’ self-perceived competency in dealing with others’ emotions is likely to be related to CC/hostility. This was not expected, but it is plausible that this may reflect a tendency for carers high on other-directed emotional self-efficacy to inaccurately, yet confidently, assume they understand service-users’ symptoms (e.g. “I understand why she is behaving in that way; I know she is staying in bed because she is lazy”). Consistent with the thesis of Barrowclough and Hooley’s [6] attributional model, this hypothesis may help to account for the observed positive associations noted between ESES ‘Dealing with Others’ Emotions’ subscale scores and CC/hostility (e.g. “I’m being critical because she needs reprimanding and encouraging”). However, it must be stated that this suggestion remains conjectural at present, and should be treated with caution, particularly given that E3 became non-significant when EOI was removed from the model.

Collectively, findings with respect to CC/hostility tentatively suggest that both attachment avoidance and facets of mentalisation may each be important therapeutic factors to consider with respect to CC/hostility, and to a roughly equal extent. Although there is a paucity of empirical data against which to compare these findings, data are consistent with attachment and mentalisation theories [13, 23], and provide support for conceptualising EE, and particularly CC/hostility, as a developmental and interpersonal process. However, it would be premature to draw firm conclusions regarding the relationships between attachment, mentalisation and CC/hostility without further research, particularly in light of the null findings with respect to the hypothesised mediation pathways, the unexpected findings with respect to other-focused emotional self-efficacy and the results of the sensitivity analysis.

No associations were observed among EOI, attachment anxiety and mentalisation, thereby refuting the hypothesis that whilst anxiously attached carers may engage in emotionally over-involved strategies in order to elicit proximity, love and support from their relative [17], mentalisation would partially mediate this relationship by facilitating sensitive and reflective caregiving [24]. As participants’ ECR:SF, RMET, ESES and FQ scores were broadly comparable with previously published literature [26, 28, 30, 34], it is unlikely, although possible, that these null findings are reflective of the participant group studied. Instead, it is possible that if associations do exist among attachment, mentalisation and EOI, then a larger sample size may be required in order for these to be detected [40].

This study has several limitations that may have influenced the generalisability of findings. First, whilst comparable with other studies using a carer population [41, 42], the current sample size rendered structural equation modelling unfeasible and resulted in one regression analysis being underpowered, therefore increasing the risk of Type II errors. Second, the paucity of available relationship data limited the potential for subgroup analyses, which may have provided further clarity on the relationships between variables. Third, the lack of conceptual clarity regarding the most effective way to operationalise and measure mentalisation means that the measures of mentalisation utilised in this study, although broad ranging, may not have fully encompassed the construct [32]. Furthermore, the low internal consistency of the RMET may have influenced the findings [43]. Fifth, diagnoses were neither confirmed nor disconfirmed, which may limit the comparability of the findings with other studies. Similarly, no measure of patient functioning was included. This was because the aim of the study was to investigate the associations among attachment, mentalisation and EE across a broad range of caring relationships; strict inclusion criteria and highly controlled conditions, removed from routine clinical practice, were therefore not felt appropriate. However, this resulted in a heterogeneous sample. Further sources of heterogeneity lie in the extent of co-morbid disorders and the levels of face-to-face contact. Clearly multiple morbidities imply greater care requirements and the impact of EE has been known to be moderated by contact times [44, 45]. Sixth, although the study follows mostly a trait logic in hypothesising relationships between attachment, mentalisation and EE, theories of attachment and mentalisation emphasise that, whilst dispositional, both attachment styles and mentalisation ability are differentially expressed according to contextual demand [23, 32, 46]. Seventh, we did not control for potentially confounding factors of the tested associations, such as severity of service users’ symptoms, whether carers and service users live together, and duration and frequency of care provision. Finally, the study’s cross-sectional nature meant that it was not possible to imply causality or direction from the findings, nor was it possible to explore changes in the observed variables or relationships over time. Furthermore, the use of self-report measures increases the risk of social desirability bias, which should be taken into account when interpreting the results.

Future studies may wish to militate against these limitations by recruiting large and representative samples of carers from clinical and non-clinical populations. Future research should aim to clarify the nature of the relationship (s) between attachment, mentalisation and EE, together with potential mediating and moderating factors such as relationship to the care recipient, illness type and severity and weekly time spent caring. Of particular interest may be the potential influence of the interaction between carers’ and service-users’/families’ attachment, given that attachment and mentalisation are interpersonal processes [47]. It may also be beneficial to consider the potential role of guilt and/or shame, given their relational nature and empirical links to both attachment [17] and EE [8]. Finally, mixed inter and intra-individual approaches which enable researchers to examine behaviour over a range of contexts, thus elucidating both stability and situational variability of carer-patient interactions, are recommended for future investigation. Telling family members that the care they give is high in expressed emotion, in some senses toxic to the person they care for, is clearly insensitive and may heighten the problem because of guilt and shame [8]. The research presented here, one way of re-conceptualising carer behaviours, may enable a more sensitive understanding of carers’ experiences, which may ultimately allow for increasingly effective support to be developed for them and the relatives they care for.

## Conclusions

Despite its limitations, the findings of this study extend current knowledge of the associations between attachment, mentalisation and EE in carers of people with long-term mental health difficulties. Specifically, the findings that carers’ attachment avoidance and specific aspects of mentalisation are each associated with levels of criticism and hostility indicate that it may be beneficial for clinicians to consider attachment and mentalisation in their conceptualisations of carers’ criticism and hostility [48]. However, it would be premature to recommend specific FIs, such as those which explicitly take into account attachment perspectives [47] and mentalisation [23], without further research to clarify the nature of the relationships between attachment, mentalisation and EE, together with their mechanisms of action.

## Abbreviations

ANOVA: Analysis of variance
CC: Critical comments
CFI: Camberwell Family Interview
ECR:SF: Experiences in Close Relationships: Short Form questionnaire
EE: Expressed emotion
EOI: Emotional over-involvement
ESES: Emotional self-efficacy scale
FI: Family intervention
FQ: Family questionnaire
IWM: Internal working model
RMET: Reading the mind in the eyes questionnaire
SPSS: Statistical package for the social sciences
ToM: Theory of mind
UK: United Kingdom
VIF: Variance inflation factor

## Footnotes

1 This approach was chosen because it was felt that participants may feel more comfortable disclosing feelings about close relationships in an anonymous, self-report context
